# Maximize Eco-Economic Benefits with Minimum Land Resources Input: Evaluation and Evolution of Land Use Eco-Efficiency of Agglomerations in Middle Reaches of Yangtze River, China

**DOI:** 10.3390/ijerph20031985

**Published:** 2023-01-21

**Authors:** Jie Zhang, Yajing Wang, Jiangfeng Li

**Affiliations:** 1 School of Public Administration, China University of Geosciences, Wuhan 430074, China; 2 College of resources, Sichuan Agriculture University, Chengdu 611130, China

**Keywords:** eco-efficiency of land use, the middle reaches of Yangtze River, Super SBM-DEA model, ecosystem services value, slacks analysis

## Abstract

Increasing land-use eco-efficiency can alleviate human-land conflict in urban areas as well as improve regional urbanization quality to achieve sustainable development. As the central urban agglomeration in China, the Middle Reaches of Yangtze River (MRYR) has experienced rapid urbanization and huge land-use change during 2000 to 2020, which poses great threats to its ecological environment. This study adopted the Super-Slack-Based Data Envelopment Analysis (Super SBM-DEA) model to evaluate the eco-efficiency of land use in MRYR. The result shows that the average eco-efficiency value of land use is above 0.77 for each year, indicating that the general efficiency is at a middle level. The trend of the evolution of the eco-efficiency can be summarized as a “U-shape” style curve. The variance between the four urban agglomerations of the MRYR changed over time. Not all capital cities or cities with higher GDP per capita obtain higher eco-efficiency in this study. Policy intervention, population and land use, technique, and environmental pollution are influencing factors of land-use eco-efficiency. Based on slacks analysis, this study proposed the optimization of the land-use structure to improve eco-efficiency from four aspects of land-use structure, investment and labor, ecosystem services value (ESV) and environment pollution, and industry structure.

## 1. Introduction

Sustainable development promotes economic growth while also taking into account the need to maintain the environment for future generations [1]. Ecological civilization is an inevitable requirement for the harmonious development of man and nature, and the construction of ecological civilization is fundamental for the sustainable development of China. According to the European Environmental Agency (EEA), eco-efficiency is the ability to maximize the benefits of fewer natural resources [2].

This can be used to gauge how resource utilization, pollution emissions, and economic growth are related [3,4]. The fact that eco-efficiency connects the environment and the economy makes it a crucial instrument for assessing sustainable development [5]. Meanwhile, regulations that encourage efficiency are more likely to be implemented than those that limit economic activity, particularly in developing countries such as China [6]. Thus, eco-efficiency research has become a hot issue in sustainable development research [7,8,9]. Urbanization has had a significant impact on the world. Large-scale land conversion is a significant issue in China, where growing urbanization has also created major land-use problems [10]. Land resources play a vital role in the ecological environment, and should be used sparingly and efficiently. The sustainable and efficient use of land resources is intimately tied to the eco-efficiency of land use. Eco-efficiency of land use can be defined as the reduction in inputs from land resources, in order to attain sustainable development goals and to achieve a mutually beneficial situation for the economy, resources, and environment. Eco-efficiency of land use cannot be quantified in absolute terms, but it does depend on the socioeconomic activities carried out on it [11]. Research on the eco-efficiency of land use in China is extremely valuable, in order to preserve land resources, safeguard the environment, and advance sustainable development.

Urban agglomeration is the area where land-use change and production activities are most concentrated. The land of urban agglomeration is a gathering place for social and economic activities, and its utilization process causes a certain degree of impact on the environment and ecosystem [7]. In East Asia, urban agglomerations have grown in Japan and South Korea since the 1950s. From the 1960s through the 1980s, South Korea relied on traditional heavy chemical industries, processing, and export industries to achieve rapid economic growth. Since the 1990s, with the disappearance of the demographic dividend and the establishment of the WTO, the domestic and foreign environments that supported South Korea’s continued rapid economic growth have undergone major changes. This has forced South Korea to achieve industrial transformation and upgrade through reform, and embark on an efficient, intensive, and environmentally friendly high-quality development path. Japan’s Tokyo Metropolitan Area and the Tokyo Bay Area, as a world-class mega city group, have brought considerable agglomeration economic effects to Japan. Compared with China, Japan’s economic center is more concentrated. The polarization of the metropolitan area is so severe that it differs too much from other cities. This enlightens China to establish a multi-dimensional urban agglomeration, to cooperate with each other and complement each other, and to form a coordinated urban agglomeration development model with coordinated industrial land use. In China, urban agglomerations mainly began to develop in the 1990s. Since the beginning of the revolution, the Yangtze River Delta (YRD) urban agglomeration, the Pearl River Delta (PRD) urban agglomeration, and the Beijing-Tianjin-Hebei (BTH) metropolitan area have been recognized as the three major growth poles of China’s economic development. The middle reaches of the Yangtze River, by building a new urbanization frontier zone in the central and western regions, can be built into a green growth pole of China. Although the socioeconomic development of urban agglomerations has achieved remarkable achievements, there are also increasing ecological and environmental threats. An extensive economy has been promoted in China for a long time [2]. In the context of this, the threat that urban agglomerations’ economic expansion poses to ecosystems has increased, making it a highly concentrated and intensifying highly sensitive area of a series of ecological and environmental problems.

In terms of research scales, most studies related to the eco-efficiency of land use have been assessed from the perspective of national and provincial levels [12,13]. However, few studies have integrated the analysis of ecosystems and socioeconomic elements at the regional scale in the context of sustainable development. Due to this, there is a lack of theoretical foundation for developing strategies and policies for urban agglomerations. In terms of research methods, at the moment, the most widely used modeling methods are stochastic frontier analysis (SFA), slack-based measurement (SBM), and data envelopment analysis (DEA). Traditional DEA methods do not account for the influence of slack variables and do not exhibit the characteristics of non-parametric statistics [14]. Traditional SFA requires the definition of a specific function of the error term consisting of a null term and a random error term, which has specification and estimation problems [15]. Because the influences of environmental and stochastic factors are not taken into consideration, traditional SBM has the disadvantage of not being able to compute the efficiency values of all decision units, and traditional SBM-DEA has the issue of bias in arithmetic efficiency [16]. In terms of research contents, previously, the studies of land-use efficiency mainly discussed the land-use efficiency of built-up land in urban areas [17,18,19,20]. In recent years, scholars have combined the study of land-use-cover change study with eco-efficiency; how to measure land-use efficiency in the context of environmentally friendly developments [21,22,23]. Eco-efficiency is not prioritized by scholars. Instead, they concentrate on either pure economic efficiency or holistic efficiency. At the same time, scholars have focused more on the eco-efficiency of a certain type of land, such as industrial land and cultivated land, or land of a certain function, for example, mining land. In fact, the land-use adjustment should consider all kinds of land-use types. This enables the analysis of the eco-efficiency of land use in light of the structural composition of the land. In terms of environmental policy, spatial planning, and other regulations, environmental impact assessment can significantly lessen the harmful effects of initiatives on the environment and contribute significantly to sustainable development [24]. The weak points of the environmental impact assessment process are frequently noted as the lack of sufficient scientific evidence in impact assessment studies and the minimal engagement of experts in policy and decision-making [25]. A crucial instrument for determining environmental sustainability in the context of assuring economic growth is the ex-ante environmental evaluation [24]. In fact, the eco-efficiency of land use is analyzed, helping to design pertinent regulations specifically in the context of pre-assessment. The need for expert knowledge and citizen participation in fostering innovation and broad adoption of strategic plans for spatial planning [26]. Starting with land-use type research on the eco-efficiency of land use can help regulate the structure of land use and offer some theoretical groundwork for the creation of pertinent spatial planning.

In this paper, we introduce ecosystem services value (ESV) as the ecological output in the process of land use and utilize the Super-SBM model. By reclassifying all types of land data into three categories-farming land, construction land, and other land-we employ all types of land data acquired from remote sensing images as the input for land resources. It can raise the value of the results, provide precise and targeted land-use adjustment targets, and help with the creation of pertinent policies.

**Figure 1:**
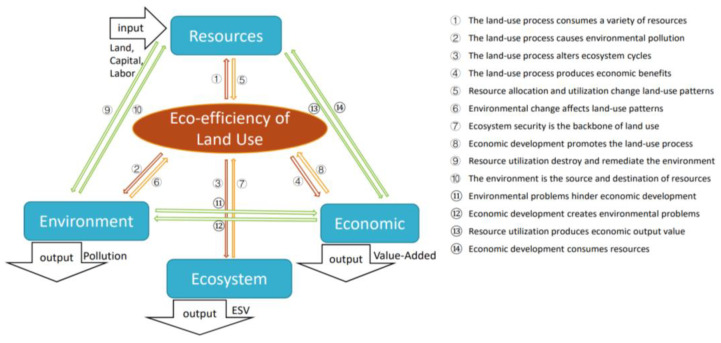
Framework of the input and output factors in the assessment of the ecological efficiency of land use. Source: own elaboration.

As shown in Figure 1, this paper builds an evaluation system with the eco-efficiency of land use as the core. The eco-efficiency of land use in urban agglomerations is centered on the input of land resources and the output of ecological and economic value. It obtains the economic value and ecological service value and the negative impact on the environment in the land-use process, and the comprehensive ability to achieve the goal of using the land resource efficiently. Improving the eco-efficiency of land use requires the coordinated development of the socioeconomic subsystem and the ecological environment subsystem.

The evaluation of land-use eco-efficiency takes three basic production factors, land, capital, and labor as the input, and environmental pollution, ecosystem service value, and economic output value as the output. Focusing on the core of land-use eco-efficiency, it first expounds the impact and interaction mechanism of the land-use process on resources, environment, ecology, and economy. Secondly, the interaction between input and output factors is analyzed. Let us take the example of a city called S. In the process of the utilization of farming land, construction land, and other natural ecological land, S city in the urban agglomeration invests labor resources and capital, and finally produces ecosystem service value and economic output value, accompanied by certain environmental pollution. The environmental pollution caused by the process changes the land-use pattern, limiting economic development and industrial upgrading.

## 2. Methodology and Data Sources

### 2.1. Study Area

**Figure 2:**
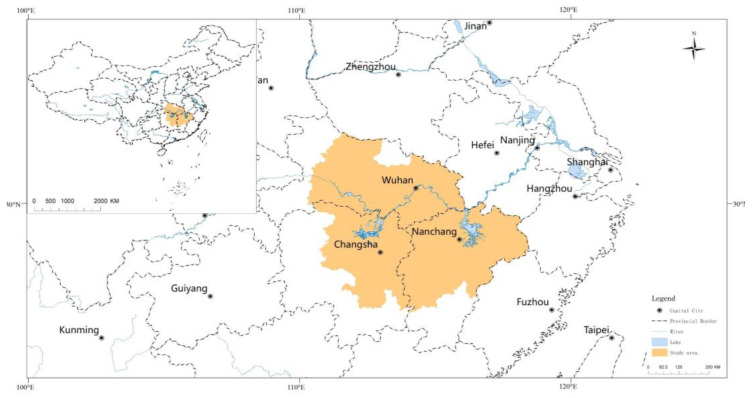
Geographical location and main cities of the middle reaches of the Yangtze River. Source: National Foundation Geographic Information Center and own elaboration.

The Yangtze River is the largest river in China and the third largest river in the world, and “The Yangtze River Economic Belt Strategy” is one of China’s national strategies. In the Yangtze River Basin, the connection between cities is relatively close, and the natural “golden waterway” can greatly reduce transportation costs (http://www.gov.cn/xinwen (accessed on 10 September 2014)). As an important part of it, the Middle Reaches of Yangtze River (MRYR) is identified as the new growth pole by the State Council of China. In the national “14th Five-Year Plan” outline, the positioning of the MRYR urban agglomeration has been upgraded to the same echelon as the Beijing-Tianjin-Hebei, Yangtze River Delta, and Pearl River Delta (http://www.gov.cn/zhengce (accessed on 15 February 2022)). It has experienced dramatic urbanization during the study period, and land for future development has become a scarce commodity. Studying the eco-efficiency of land use is crucial for the sustainable development of the study area as well as the entire nation. As can be seen from Figure 2, this paper takes the four city groups of the MRYR urban agglomerations, which contains 31 prefecture-level cities as the study area. In the year 2020, the study area contributes about 9.3% of GDP (“Bulletin of Statistics for national economic and social development (2020)”). With a total land area of 326,000 square kilometers, accounting for 3.4% of the country, it is the largest urban agglomeration in China, 1.5 times that of the Yangtze River Delta and 6 times that of the Pearl River Delta (China Urban Statistical Yearbook 2018). The permanent population is about 130 million people, accounting for 9.1% (http://www.gov.cn/zhengce (accessed on 15 February 2022)) of the country, only lower than the Yangtze River Delta.

(1) Wuhan City Circle and Xiang-Jing-Yi City Belt

The Wuhan city circle includes “1 + 8” cities, which are Wuhan and Huangshi, Ezhou, Huanggang, Xiaogan, Xianning, Xiantao, Qianjiang, and Tianmen around it. The natural environment of the land in the Wuhan city circle is diverse and the land resources are distributed in a multi-level circle. Same in the Hubei province. The Xiang-Jing-Yi City Belt includes Xiangyang, Jingmen, Jingzhou, and Yichang. It is an urban economic development belt in western Hubei formed by the Jiaoliu Railway, E’guang Expressway, and Hanjiang River. The two city groups use 64.6% land area contributing up to 90.9% GDP of Hubei province in 2020 in Table 1.

(2) Poyang Lake City Circle

The city cluster around Poyang Lake covers 10 cities including Nanchang, Jingdezhen, Yingtan, Jiujiang, Xinyu, Pingxiang, Fuzhou, Yichun, Shangrao, and Ji’an. The land area is 128,662 square kilometers, which occupies 77.1% of the land area of Jiangxi Province. The GDP accounts for 85.8% of Jiangxi Province. The infrastructure construction of the Poyang Lake city circle has been continuously improved, and the urban layout structure has basically formed.

(3) Chang-Zhu-Tan City Circle

The Chang-Zhu-Tan City Circle consists of 8 cities of Changsha, Zhuzhou, and Xiangtan, and the surrounding Changde, Yiyang, Yueyang, Hengyang, and Luodi. The distances between Changsha and Zhuzhou are 49 km away, Xiangtan and Zhuzhou are 37 km away, and Changsha and Xiangtan are 51 km away. The layout of the three medium-sized cities is compact, and the transportation between the cities is very convenient. The total land area of the Chang-Zhu-Tan City Circle is about 97,606 square kilometers, accounting for 46% of the province’s land area.

### 2.2. Data Sources

The research data of this paper come from “Geospatial Information Platform of Chinese Academy of Sciences”, “China Urban Statistical Yearbook 2001–2020”, “China Urban Construction Statistical Yearbook 2001–2020”, “The Middle Reaches of Yangtze River Development Plan 2015”, and other provinces and cities’ statistical yearbooks and various annual reports from the government websites. The spatial dataset is from Geospatial Information Platform of Chinese Academy of Sciences. The original remote sensing image with 30 m accuracy of Landsat TM is from the United States Geological Survey (USGS) of Jiangxi Province, Hunan Province, Hubei Province. Remote sensing interpretation derived from the National Fundamental Geographic Information Systems in China.

### 2.3. Super SBM-DEA Model

At present, DEA models are the primary methods to measure land-use efficiency; the evaluation methods mainly focus on DEA models, and SFA [21,23,27,28,29,30]. They are a non-parametric efficiency estimation method that does not need the specific form of production frontier and are easier to deal with when using multiple outputs. DEA provided by Charnes et al. in 1978 [31] was based on the relative efficiency of similar decision unit multi-index evaluation of the concept of input and output efficiency of a linear programming model, known as the “Charnes-Cooper-Rhodes (CCR)” model [31]. After updating the model for variable returns to scale the “Banker-Charnes-Cooper (BCC)” model [32]. The two measures with the efficiency of the unit are based on the radial and angular dimensions, when non-zero slack calculation results will be large. Tone (2001) [33] proposed the non-radial slack-based measure (SBM) model, which can deal with the redundancy problem of unexpected output. Effectively handle the input factors “crowded” or “relaxation” phenomenon. The efficiency of the SBM model calculated the maximum value to be 1; when there are a number of cities at the efficiency of 1, it will not be able to further determine which cities are more efficient. Tone (2002) [34] solved the problem with the Super Efficiency SBM model. The model can be described as follows:

(1) $$\begin{array}{c} \;\rho =\min\;\frac{1\;-\;\frac{1}{m}\;\sum_{i=1}^{m}\frac{s_{i}^{-}}{x_{i0}}}{1+\frac{1}{k}\;\sum_{r=1}^{k}\frac{s_{r}^{+}}{y_{r0}}}\\ s.t.\;x\;\geq {\;X\lambda +s}_{}^{-},\;y0\;\leq {\;Y\lambda -s}_{}^{+},\\ \lambda \;\geq {\;0,\;s}_{}^{-}\;\geq {\;0,\;s}_{}^{+}\;\geq \;0 \end{array}$$

where ρ means efficiency value evaluation of standard; input vector $x_{0}$ has m kind of input elements, its elements, respectively, for $x_{i0}=(\;i=1,\;2,\ldots ,\;m\;)$; output vector y0 has k kind of output elements, its elements, respectively, for $y_{r0}=(\;i=1,\;2,\ldots ,\;k\;)$; X and Y, respectively, for input elements matrix and output elements matrix; $s_{}^{-}$ stands for input redundant, its elements for $s_{i}^{-}=(\;i=1,\;2,\ldots ,\;m\;)$, $s_{}^{+}$ stands for output shortage, its elements $s_{r}^{+}=(\;r=1,\;2,\ldots ,\;k\;)$. When ρ ≥ 1, the production unit is fully effective. When ρ < 1, there is loss in the DMU, which can be improved by optimizing the input and output factors.

To consider the undesirable output factors, scholars have extended the above model to divide the output vector into desired output a and undesired output b, the input vector $x\;\in {\;R}_{}^{m}{,\;\mathrm{the}\;\mathrm{desired}\;\mathrm{output}\;y}_{}^{a}\in {\;R}_{}^{a}{,\;\mathrm{the}\;\mathrm{undesired}\;\mathrm{output}\;y}_{}^{b}\;\in {\;R}_{}^{b};$ the input element matrix, the desired output matrix, and the undesired output matrix are ${X=[\;x}_{1},\ldots {.,x}_{n\;}]\in {\;R}_{}^{m\times n}{,\;Y}^{a}{=[\;y}_{1}^{a},\ldots {.,\;y}_{n}^{a}\;]\in \;R_{}^{a\times n}{,\;Y}^{b}=[\;y_{1}^{b},\ldots .,y_{n}^{b}\;]\in {\;R}_{}^{b\times n}$, assuming that ${X,\;Y}^{a}{,\;\mathrm{and}\;Y}^{b}$ are greater than zero, and the production possibility set is defined under CRS as: $P=\left\{{(\;x,\;y}^{a},\;y^{b}\;)|\;x\;\geq {\;X\lambda ,\;y}^{a}\;\leq {\;Y}^{a}{\lambda ,\;y}^{b}\;\geq {\;Y}^{b}\lambda ,\;\lambda \;\geq \;0\right\}$, the SBM-undesirable model:

$$\{\begin{array}{c} \rho_{}^{*}=\min\;\frac{1\;-\frac{1}{m}\sum_{i=1}^{m}\;\frac{s_{i}^{-}}{x_{i0}}}{1+\frac{1}{a+b}[\sum_{r=1}^{a}\;\frac{s_{r}^{a}}{y_{r0}^{a}}+\sum_{r=1}^{b}\;\frac{s_{r}^{b}}{y_{r0}^{b}}]}\\ s.t{.\;x0=X\lambda +s}_{}^{-}{,\;y}_{r0}^{a}{=Y}_{}^{a}{\lambda \;-\;s}_{}^{a}{,\;y}_{r0}^{b}{=Y}_{}^{b}{\lambda +s}_{}^{b}\;,\\ \lambda \;\geq {\;0,\;s}_{}^{-}\;\geq {\;0,\;s}_{}^{a}\;\geq {\;0,\;s}_{}^{b}\;\geq \;0 \end{array}$$

where $\rho_{}^{*}$ denotes the efficiency value evaluation criterion; the input vector x0 has m input elements, whose elements are $x_{i0}=(\;i=1,2,\ldots ,m\;)$; the desired output vector $y_{0}^{a}$ has a few kinds of output elements, whose elements are $y_{r0}^{a}=(\;r=1,\;2,\ldots ,\;a\;)$; the undesired output vector $y_{0}^{b}$ has b kinds of output elements, whose elements are $y_{r0}^{b}=(\;r=1,\;2,\ldots ,\;b\;)$; $s_{}^{-}$ denotes input redundancy, whose elements are $s_{i}^{-}=\left(\;i=1,\;2,\ldots ,\;m\;\right){,\;s}_{}^{a}$ denotes desired output deficiency, whose elements are $s_{r}^{a}=\left(\;r=1,\;2,\ldots ,\;a\;\right){,\;s}_{}^{b}$ denotes non-desired output redundancy with elements $s_{r}^{b}=(\;r=1,\;2,\ldots ,\;b\;)$. Similarly, when $\rho_{}^{*}\;\geq \;1$, then we have $s_{}^{-}{=0,\;s}_{}^{a}{=0,\;s}_{}^{b}=0$, indicating that there is no input and non-expected output redundancy and expected output deficiency, i.e., the decision unit is valid. When $0\;<{\;\rho }_{}^{*}\;<\;1$, then, the decision unit is inefficient and can be improved by optimizing inputs and outputs.

### 2.4. Calculate the ESV

In 1997, Costanza et al. [35] divided ecosystem service functions into 9 items, and calculated the total value and each sub value of ecosystem services worldwide. Their study focuses can be summed up as follows: Firstly, classify the ecosystems according to certain classification standards, such as different environment and land type of the study area. Secondly, calculate the ecosystem service value of each ecosystem according to various standards and methods. Finally, summarize the ecosystem service value of the study area. Obtain the general structure table in the area. The specific model is as follows:

(2) $$V_{t}=\overset{n}{\underset{i=1}{\sum }}\overset{n}{\underset{j=1}{\sum }}\;S_{i\;}{\times \;M}_{\mathrm{ij}}$$

where $V_{t}$ represents the total value of ecosystem services in the region in year t (CNY); $S_{i}$ represents the area of i land use type (hm^2^); $M_{\mathrm{ij}}$ stands for coefficient of class j ecosystem service function of class j ecosystem (CNY/hm^2^); i represents the numbers of land-use types; j stands for numbers of ecosystem services.

In 2008, Xie Gaodi et al. [36] formulated the research results of Costanza et al. and updated the calculation table combined with the geographical characters of China to make it more suitable for the country. They conducted a questionnaire survey of 700 professionals with ecological backgrounds in China in 2002 and 2006 to derive a new ecosystem service valuation system. The comparison showed that the expert knowledge-based ecosystem service unit price system obtained from the survey was more comparable with the quality-based ecosystem service value. This expert knowledge-based ecosystem service valuation system can be used for known land-use areas, and can obtain more accurate results in a shorter period of time. 

The model they worked out is the initial model, according to the regional actual and time-scale grain unit price; the total value of regional ecosystem services is calculated.

The model was based on research conducted nationwide. However, there are variations in the geographical environment and vegetation growth in different regions. In this study, we use the net primary productivity (NPP) of vegetation to make regional adjustments. The adjustment process is as follows:

(3) $$P_{\mathrm{ij}}=\frac{B_{\mathrm{ij}}}{B_{\mathrm{average}}}$$

where $P_{\mathrm{ij}}$ is NPP space-time regulators, $B_{\mathrm{ij}}$ refers to NPP of the j month of the i region of this type of ecosystem, and $B_{\mathrm{average}}$ refers to NPP of the annual average of this ecosystem nationwide.

### 2.5. Index Selection

The input and output indicators of economic, social, and ecological aspects related to land utilization are selected in Table 2 in order to construct a comprehensive and objective evaluation of the land-use eco-efficiency of the urban agglomerations in the middle reaches of the Yangtze River. There are 3 aspects of input: (1) Land resources, which are divided into 3 types: farming land (FAR), construction land (CON), and other land (OTH). (2) Capital resources (CAP), we choose the investment in fixed assets to measure the capital input on the land. (3) Labor resources (LAB), we choose the number of people employed in the whole society to measure the labor input on the land. At the same time, the paper chooses 3 aspects of output: (1) Ecological value (ESV), the paper calculates the Ecosystem Service Value using a certain estimate method, using it to describe the output of the land use. (2) Economic value, the added value of the first (FIR), secondary (SEC), and tertiary (TER) industries are selected. (3) Environmental pollution, the paper selects industrial sulfur-dioxide emissions (SO_2_) and industrial wastewater discharge (WAS) according to the principles of data availability and precision.

## 3. Results

### 3.1. Isotonicity Analysis

**Figure 3:**
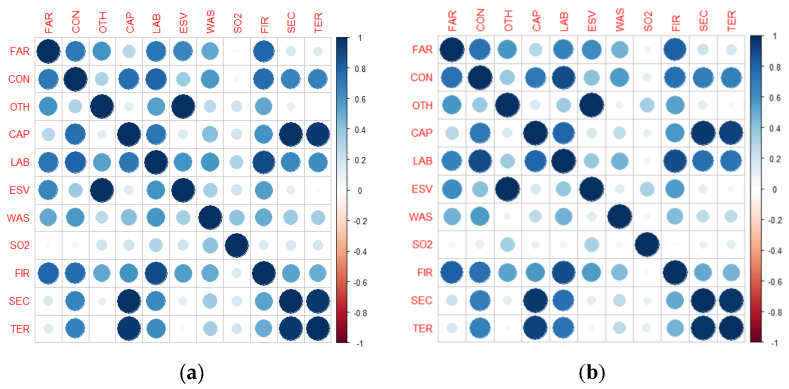
Correlation of inputs and outputs of 2015 (**a**) and 2020 (**b**).

Before using DEA models, it is necessary to test whether the data meet the assumptions of the models. First, the number of units should be at least twice the number of inputs and outputs. The number of DMUs in this study was much larger than the number of inputs and outputs, which met the quantity conditions. In addition, the input-output indicators must satisfy the isotonicity assumption that an increase in any input should not result in a decrease in any output. In this study, the Pearson correlation coefficient was used for testing (Figure 3). The correlation coefficient table shows that all input-output indicators passed the isotonicity test. Thus, the constructed indicator system can be analyzed using DEA models.

### 3.2. Temporal-Spatial Trends of Land-Use Eco-Efficiency

#### 3.2.1. Land-Use Eco-Efficiency of four City Groups

- Wuhan City Circle and Xiang-Jing-Yi City Belt

**Figure 4:**
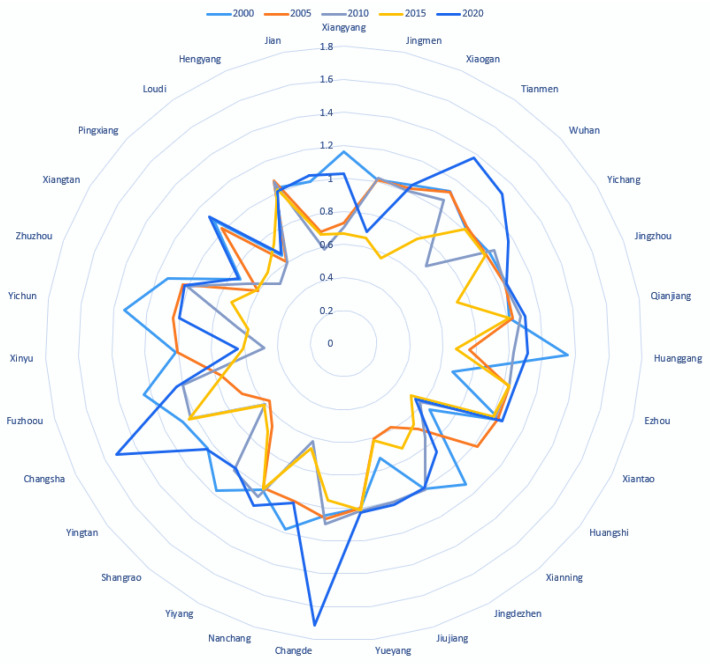
The results of the Ecological Efficiency of land use.

Wuhan City Circle and the Xiang-Jing-Yi City Belt are both located in Hubei Province, which is the geographically central province of China. Wuhan, the capital city of the province, is known as the “thoroughfare of nine provinces”. From Figure 4, it can be seen that Wuhan’s eco-efficiency of land-use level improved after 2010, when it was lower due to the excessive pollution emissions and insufficient agricultural production values. Wuhan’s eco-efficiency of the land-use level was at the middle level in the province in 2000, 2005, and 2015, especially, in 2020 when it reached a higher level among all cities in the study area. Huangshi’s eco-efficiency of land use has the lowest value of eco-efficiency of land use in Hubei province, and the large input of labor and land resources without the corresponding economic output made its value of eco-efficiency of land use at the lowest level in most years. Qianjiang, Tianmen, and Xiantao, as the three county-level cities under the province’s administration, maintained high-efficiency levels from 2000 to 2020, mainly due to various land-use policies and the development of green industries. Tianmen City, with its ecological environment and other advantages, was at a high level of efficiency value except for 2010, and reached the maximum provincial efficiency value in 2020. The efficiency values of Huanggang and Xianning show a “W-shaped” trend. From 2000 to 2005, the eco-efficiency of land use in Huanggang and Xianning showed a sharp downward trend, as the area of construction land increased dramatically while the ecological and economic output remained constant. From 2015 to 2020, the efficiency values showed an upward trend attributable to the industrial reorganization from high-polluting industries to tourism. Ezhou city showed an upward trend in eco-efficiency of land use during the study period, which indicates that the city’s land-use is developing toward rationalization and high-efficiency with high-quality.

In the Xiang-Jing-Yi urban belt, Xiangyang city has a low level of eco-efficiency of land use and shows a “U-shaped” trend of first rising and then falling, which is due to the fact that Xiangyang’s production factor inputs are at a medium level while its economic output is at a low level. Jingmen City shows a decreasing trend in general, whereas Jingzhou City and Yichang City show a medium level overall; this is because these cities have more land input than other cities in the province, but insufficient economic output.

- Poyang Lake City Circle

In Poyang Lake City Circle, Nanchang, the capital of Jiangxi Province, had a higher value of eco-efficiency of land use in 2000 and a lower value of eco-efficiency of land use ranking afterward, which may be due to the waste of land caused by the blind expansion of the city and the environmental pollution caused by the unreasonable industrial structure since 2000, which has further exacerbated the decrease in land-use eco-efficiency until 2020. Fuzhou, Shangrao, and Yichun converge with Nanchang. The eco-efficiency of land use in Jingdezhen, Ji’an, Yingtan, and Pingxiang first decreases and then increases steadily in a “U-shaped” trend, which may be explained by the region’s low economic growth. The eco-efficiency of land use in Xinyu city declined and remained at a low level during the study period. The eco-efficiency of land use in Jiujiang City showed a general upward trend and decreased in some years, mainly owing to the same problem of insufficient economic output, which led to the lack of obvious economic benefits of land inputs.

- Chang-Zhu-Tan City Circle

In the Chang-Zhu-Tan City Circle, Changsha, as the capital of Hunan province, is the national “two-type society” comprehensive supporting reform pilot area. From Figure 4, we can see that the value of eco-efficiency of land use in Changsha City has been on the rise since 2005 during the study period, until 2020, when Changsha City’s value of eco-efficiency of land use was second only to Changde City, reaching an efficiency value of 1.52. This is closely related to the fact that Changsha and Changde do not blindly pursue urban expansion and have a friendly development policy orientation. Zhuzhou City, Hengyang City, Yiyang City, and Yueyang City all maintain high levels of land-use eco-efficiency. Loudi and Xiangtan cities have been at a low level of land-use eco-efficiency value, and according to statistical data, their economic output value is insufficient as the main reason.

#### 3.2.2. Trends of the Eco-Efficiency of Land Use

**Figure 5:**
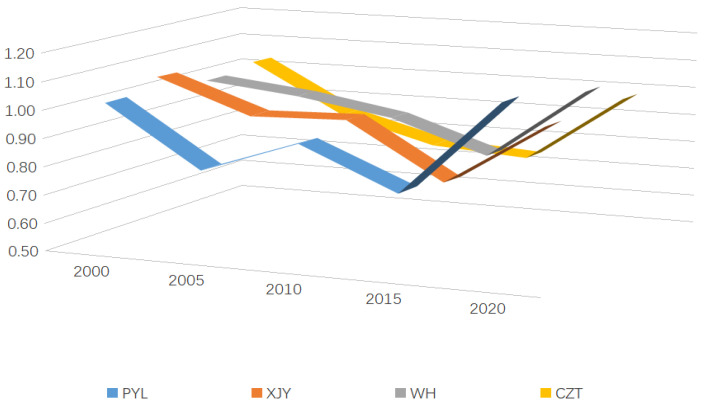
Average eco-efficiency of 4 urban agglomerations.

From Figure 5, the middle reaches of the Yangtze River urban agglomeration have average annual land-use eco-efficiency values over 0.77, which indicates that the overall efficiency is in the middle to upper range. The eco-efficiency of land use in MRYR shows a decreasing and then increasing trend, which we summarize as a “U-shaped” curve. From the perspective of urban clusters, the average eco-efficiency of four urban clusters in MRYR is ranked as WH (average 0.95) > XJY (average 0.94) > PYL (average 0.93) > CZT (average 0.87). In 2000, XJY had the highest average efficiency value and WH had the lowest; in 2005, WH had the highest value and PYL Circle had the lowest. PYL and XJY show “W-shaped” curves from 2000 to 2020, twice experiencing a rise and fall. WH and CZT show “U-shaped” curves, with higher eco-efficiency of land use in 2000 and 2020, and lower eco-efficiency of land use in 2005, 2010, and 2015.

#### 3.2.3. Focused Cities’ Eco-Efficiency of Land Use

**Figure 6:**
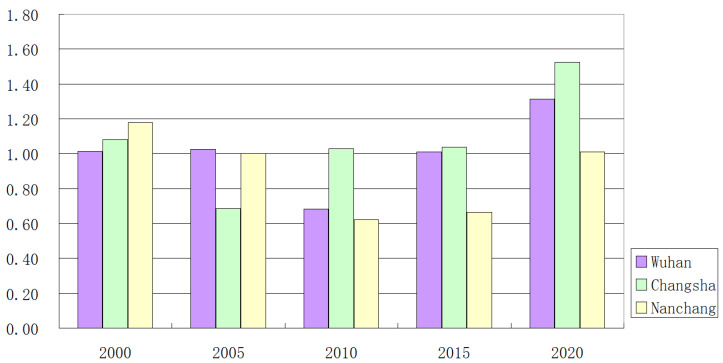
Capital cities’ eco-efficiency of land use.

From the perspective of cities in Figure 6, the cities with the highest eco-efficiency of land use are, in order, Huanggang (2000), Tianmen (2005), Changde (2010), Changsha (2015), and Changde (2020), and the cities with the lowest eco-efficiency of land use are Loudi (2000), Yingtan (2005), Xinyu (2010) Huangshi (2015, 2020). During 2000-2005, most cities showed a decreasing trend, and the eco-efficiency of land use of Ezhou City increased the most from 0.67 to 1.03; the eco-efficiency of land use of Huanggang City decreased the most from 1.35 to 0.76. During 2005-2010, most cities showed an increasing trend, and the eco-efficiency of land use of Jingdezhen City increased the most from 0.57 to 1.01; the eco-efficiency of land use of Xinyu City decreased the most, from 1.00 to 0.47. From 2010 to 2015, most cities show a decreasing trend, with Wuhan City’s eco-efficiency of land use increasing the most from 0.68 to 1.00, and Xiaogan City’s eco-efficiency of land use decreasing the most from 1.00 to 0.56. From 2015 to 2020, most cities show an increasing trend. The eco-efficiency of land use in Changde City increased the most from 0.95 to 1.71, and the eco-efficiency of land use in Loudi City decreased the most from 0.73 to 0.66. Among the provincial capital cities, Changsha has a “U-shaped” trend with the highest mean value; Wuhan has a “V-shaped” trend with the second highest mean value; and Nanchang has a “W-shaped” trend with the lowest mean value. The provincial capital cities have bottlenecks regarding the problem of how to use land resources efficiently and ecologically, which will be improved to some extent in 2020 under a series of policy interventions and other influencing factors.

### 3.3. Influencing Factors

#### 3.3.1. Policy Summary

China pursues comprehensive and coordinated sustainable development, and ecological safety in the Yangtze River basin is of increasing concern. From 2000 to 2005, efficiency values dropped significantly. The rapid growth of GDP at this stage relied on the high consumption of resources, resulting in high environmental pollution. At the same time, all kinds of environmental problems broke out intensively, and environmental protection became an important trigger point for social conflicts of interest.

In 2002, the report of the 16th National Congress of the Communist Party of China put forward the goal of building a well-off society in an all-around way, requiring the continuous enhancement of sustainable development capabilities, the improvement of the ecological environment, and the significant increase in resource utilization efficiency. In 2017, the 19th National Congress of the Communist Party of China proposed high-quality economic development and other environmental regulating measures, China’s economy is progressively transitioning to high-quality intense development. Among these policies, the ecological compensation policy is an important one, aiming at protecting the ecological environment and using economic instruments to coordinate the interests of stakeholders in favor of sustainable development. Ecological compensation will be implemented in terms of environmental pollution, economic status, and ecosystem values, which are closely linked to the measurement of eco-efficiency of land use. In the context of China’s ecological compensation policy, Dong [15] measured the eco-efficiency of land use in small watersheds, and the results showed that the efficiency gradually improved under the first round of ecological compensation policy implementation.

#### 3.3.2. Population and Land Use

With the rapid advancement of the urbanization process, urban development has attracted more and more attention from society. Most local governments equate “urbanization” with “urban construction”, placing too much emphasis on the growth of built-up areas at the expense of urban population clustering and social security implementation after clustering. From 2000 to 2011, the area of urban built-up areas increased by 76.4%, which was much higher than the growth rate of the urban population of 50.5%, and “land urbanization” was significantly faster than “population urbanization”. Continuous urbanization has brought about a number of issues, including deteriorating environmental quality, a lack of cultivated land, and deterioration of ecosystem processes, which have altered the structure and operation of regional ecosystems [40]. The results of Shan’s [41] research show that large population living in cities, increasing urban population density, and the demand for resources from city dwellers all harm the environment and lower efficiency levels.

The urban-rural dualist system, economic growth, and land-use regulations are all connected with land-use concerns in China, which have distinctively Chinese characteristics. Large-scale land conversion due to rapid urbanization has led to major issues with land use, including the loss of cultivated land, the abandonment of cultivated land, and the formation of hollow villages, which pose a threat to China’s resources and food security. As the carrier of urban social, economic, political, and cultural activities, urban land is the spatial basis for the realization of the overall function of the city. In the process of urban development, the fact that the scale of land use has expanded too fast, the extensive use of construction land, and the continuous erosion of cultivated land and ecological land have brought serious consequences to urban construction and social and economic development, making the already scarce land resources suffer. With large-scale enclosures such as “new districts”, “new towns”, and “development zones”, the contradiction between land-use supply and demand has become more prominent, the demand for new construction land is large, and the situation of cultivated land protection is severe. Land-use conditions can have different effects on the eco-efficiency of different areas [11]. In our study, land resources were divided into three categories of farming land, construction land, and other land, and different land-use types were used as input factors to explore the eco-efficiency of land use. According to Lu [42], cultivated land resources are both a source of agricultural products and a significant source of carbon emissions, which can have an impact on the stability of regional ecosystems. This shows that the ecological efficiency of land use is significantly influenced by land use.

#### 3.3.3. Techniques and Social Factors

In China, energy savings and emission reductions have gained considerable attention due to environmental pollution and excessive resource consumption caused by rapid economic growth and urban expansion. Since entering the 21st century, China’s clean energy has developed rapidly. The installed capacity of hydropower, solar thermal utilization, wind power, and solar power generation have successively become the first in the world. As of the end of 2018, the proportion of hydropower in China’s installed capacity of clean energy had dropped from 100% in 1949 to 45.52%, while the installed capacity of wind power had reached 23.81%, the installed capacity of photovoltaic power generation had reached 22.57%, the installed capacity of nuclear power had reached 5.77%, and the installed capacity of biomass power generation had reached 2.3%. The development and expansion of terminal applications have also led to the comprehensive development of China’s clean energy industry chain. According to Yang [43], higher levels of research and development can help facilitate innovation in cleaner production methods, accomplish cleaner production at the source, lower the intensity of resource consumption, and produce more of the desired output while decreasing more of the undesirable output. This shows that improvements in land-use eco-efficiency are facilitated by developments in science and technology.

Environmental pollution is a major challenge to the sustainable development of human societies and natural ecosystems. Carbon emissions have gained a lot of attention from people all over the world. Agricultural carbon sources, such as the generation of agricultural waste, rice cultivation, and the burning of biological tissues, are directly related to land-use activities [44]. Kuang [13] explores the efficiency of provincial cultivated land use in China, using carbon emissions as an undesirable output. Dong [15] measures the efficiency of chemical elements such as lead, total phosphorus, fluoride, and selenium while taking into account pollution from home sewage and fertilizers. Environmental pollution is an important factor affecting the eco-efficiency of land use.

### 3.4. Slacks Analysis and Optimization Adjustment

The paper calculated the eco-efficiency of land use of the study area and briefly analyzed the previously reported potential influence factors. Based on that, this paper summarizes the slacks of the inputs and outputs of the model, to figure out the targeted constraints to the eco-efficiency of land use of the city groups. The Super-SBM model is used in the evaluation of the eco-efficiency of land use. When measuring efficiency, the DEA model is evaluated according to the input and output levels of each decision-making unit. The efficiency value that is measured is relative efficiency; the level of efficiency in comparison to the most efficient decision unit [45]. Therefore, cities not found to be DEA effective may have constraints from inputs, outputs, or both inputs and outputs. Those are factors that limit efficiency improvement. The variables that constrain efficiency gains in the data envelopment model are referred to as slacks, including “input redundancy” and “output insufficient”. “Input redundancy” means the quantity of inputs that can be saved from the associated resources in order to attain efficiency; “output insufficient” means the number of outputs that must be increased in order to achieve efficiency; in this article, we found that the corresponding ecological and economic values can be improved and the potential for pollutions can be reduced. The input and output factors can be seen in Table 3; the paper takes the first three letters to give a short-term for the factors in this analysis part. This part is to analyze the slacks, and make progress on the input and output of each city group on this basis. Scientific and reasonable optimization is conducive to realizing the most optimal land use structure.

#### 3.4.1. Land-Use Structure

In terms of input factors, all urban agglomerations have some degree of redundancy in most years. In terms of land resources, there is a surplus of agricultural land, construction land, and other land from 2000 to 2020, which indicates a certain degree of waste in the use of land resources. However, it differs from one urban agglomeration to another in different years. For the year 2000, the main land structure problem in the Xiang-Jing-Yi City Belt is the excess input of agricultural land and construction land. For WH, the main problem is the excess input of construction land. For CZT, the main problem is the excess input of agricultural land as well as ecological and other land. For PYL, the main problem is the excess input of ecological and other land. The four agglomerations all experienced redundancy in 2005 and 2010. In 2015, there was some redundancy of agricultural land and construction land in PYL, XJY, and WH, and redundancy of agricultural land and ecological and other land in CZT. In 2020, there was overcapacity in agricultural land and construction land in XJY, and there was input overcapacity in other land in Wuhan City Circle. The results show that most cities in the middle reaches of the Yangtze River urban agglomeration have a certain degree of unreasonable land-use structure, with the most prominent problem of non-intensification of construction land. Among them, 45% of the cities had different degrees of redundancy of construction land in 2005. The comparative analysis shows that the redundancy rate of construction land differs among cities, but there is a certain commonality in the land-use structure adjustment programs between provincial capital cities and cities in urban areas.

#### 3.4.2. Investment and Labor Force

In terms of fixed asset investment, excluding XJY and PYL in 2005 and WH and XJY in 2015, there is some redundancy in all four urban clusters in other study years. In terms of employment, most of the urban agglomerations have labor redundancy, excluding XJY in 2000. In 2000, the capital and labor redundancy in CZT is at a high level in comparison. Within the Chang-Zhu-Tan City Circle, redundancy in fixed asset investment is concentrated in Changsha, Zhuzhou, and Loudi, and redundancy in the number of employees is concentrated in Loudi and Xiangtan. In 2005, WH shows significant redundancy in investment and labor force, but there are differences among cities. The only cities having redundancy in fixed asset investment are Wuhan and Tianmen, and the only cities having redundancy in the labor force are Huanggang and Xianning. In 2010, the redundancy was mainly concentrated in CZT. In 2015, the redundancy of the labor force was mainly concentrated in Wuhan City Circle and Poyang Lake city circle; meanwhile, the redundancy of capital investment was mainly concentrated in PYL. In 2020, there is no obvious redundancy of labor force input in most of the city groups, and the urban development and ecological efficiency of land use improve to absorb the surplus labor force. However, there was a large surplus of fixed capital input in that year, which may have been due to the influence of the high percentage of land finance.

#### 3.4.3. Ecological Output and Environmental Pollution

During the years of the study area, all four urban agglomerations included in the middle reaches of the Yangtze River urban agglomeration had the problem of insufficient ESV output, indicating that further land use control is needed to protect the ecological environment and enhance the service function of the ecosystem. Statistically, most cities in the study area had serious industrial SO_2_ emissions and industrial wastewater discharge pollution in most of the years from 2000 to 2010. After 2015, the pollution was greatly modified. In terms of output insufficiency analysis, industrial wastewater discharges in the four metropolitan agglomerations are currently within a reasonable range and have no discernible effects on the improvement of land-use eco-efficiency.

#### 3.4.4. Industrial Structure

During the study years, the shortage of economic output value was mainly concentrated in the tertiary industry. The amount of redundancy of tertiary industry output differs among the four urban groups. If the optimization results of the DEA model can be realized, in 2020, the output value of the primary industry can be increased by CNY 147.717 billion, the secondary industry by CNY 571.810 billion, and the tertiary industry by CNY 113.884 billion, which is equivalent to about 2% of the total GDP of China in that year. Industrial upgrading is an issue that must be considered to enhance the ecological efficiency of land use.

## 4. Discussion

The paper here proposed some suggestions and recommendations for land-use management based on the study results above.

The paper discussed the changes in land-use eco-efficiency during the years 2000 to 2020; we summarize the land-use-cover change, industrial structure change, pollution, etc., to analyze the reasons. These results point us in the direction of improvement in the green and efficient use of land resources.

Sustainable development should be emphasized throughout the entire decision-making procedure [46,47]. The government should take activities to deal with the conflicts between land use and the ecological environment [30]. It is an important guarantee for economic development and security of food rations through the combination of land use and land cultivation, the utilization of cultivated land guaranteeing the current production.

The improvement of eco-efficiency of land use should be set as one of the core factors of local government performance evaluation. The improvement of urban eco-efficiency in China depends largely on the further reforming of performance evaluation mechanisms [48]. During the process, the eco-efficiency of land use needs to be improved according to local conditions, and the four city groups in the MRYR have their own characteristics. Cities should be based on their own functional positioning, economic vitality, and resource endowments determining their land use layout [49,50], fully tapping the potential of all kinds of land, to avoid blindly expanding the city. At the same time, the thresholds should be used to limit high energy consumption and high pollution.

According to the land use and industrial layout of different cities, the division of labor and cooperation will be carried out to advance industrial upgrading. Industrial upgrading is an important way to improve the level of eco-efficiency, which is a key factor affecting energy consumption intensity and pollution emission intensity [51].

This study has some deficiencies, first of all, there is no temporal continuity in the land-use data, so the efficiency is measured intermittently. As a result, the calculated efficiency value cannot reflect the trend of change and spatial differentiation patterns, and the inflection point of efficiency values cannot be determined. Secondly, the idea of eco-efficiency only makes sense when viewed through the lens of sustainable development. Meanwhile, this article is based on the city level, which cannot take into account the differences within the region. Further study can be conducted at the county level to discuss the efficiency of land-use structures to improve the present practical guidance. Third, as the number of decision-making units increases, the system of input-output indicators could also be appropriately expanded to allow for more perspectives to consider the benefits of land use and comprehensively measure the structure of land-use efficiency. Last, but not least, a comprehensive comparison of the input and output status of the land should be made on the basis of efficiency. It is more appropriate to evaluate the effectiveness of land use by considering the negative impact of the land-use process on the environment.

## 5. Conclusions

China’s high-quality development policy has required high-efficiency of land use, and it is of great significance to ensure green development while improving land-use efficiency. Therefore, the concept of achieving eco-efficiency should be widely used in land-use management. The paper constructed an evaluation system of land-use eco-efficiency of the urban agglomeration in MPYR using the Super-SBM model based on previous studies of the urban-land-use efficiency of various regions of China. After the analysis of the results of the eco-efficiency of the four city groups of the MRYR, the paper briefly summarized the changes in policy, population, land use, techniques, and social factors during the study period and how they possibly contribute to the evaluation results. At last, the paper analyzed the slacks of the input and output factors.

During the years 2000 to 2020, the eco-efficiency values are generally in a relatively upper-middle average. There exists an upside potential of improving the eco-efficiency in many cities. Each city’s efficiency varies and is heterogeneous, yet there is a general trend of falling and then rising. Most of the high-efficiency scores occur between 2000 and 2020.

The variance between the four city groups of the MRYR urban agglomeration changed every year. In 2000, the highest average efficiency value is XJY, and the lowest is WH; In 2005, the highest is WH, and the lowest is PYL Circle. PYL and XJY experienced two ups and downs from 2000 to 2020 as they move along a “W-shaped” curve. WH and CZT show “U-shaped” curves, with higher land use eco-efficiency in 2000 and 2020, and lower land use eco-efficiency in 2005, 2010, and 2015.

Not all capital cities or cities with higher GDP per capita had higher eco-efficiency in this study. Cities such as Nanchang, Changsha, and Wuhan, as the capital of their provinces, are also the economic center of the central region, with good location conditions and a large proportion of construction land. However, their eco-efficiency turns out to be at an average level for some years because of their environmental problems and extensive land-use pattern. Although some of the relatively developed cities have the highest pollution, their economic output has an absolute advantage, making their eco-efficiency still reach the optimal level. Some economically underdeveloped areas also showed high land-use levels, such as Pingxiang and Yiyang, because of their high level of green development. Some cities are in the middle level of both economic and the eco-efficiency, which may be because the advantages of the urban economy in this study are not very prominent, but in the process of development, extensive land use has produced higher pollution emissions, and undesirable environmental output has pulled down the overall land-use level.

In this paper, the main ecological protection policies in the research period are sorted out and the influencing factors of the policies are analyzed qualitatively. Considering China’s national conditions, the promulgation and implementation of policies have a certain time consistent with the changes in the eco-efficiency of urban agglomerations. In addition to government factors, population, land-use patterns, technical progress, etc., may have significant effects on efficiency values.

The slacks analysis can be used to optimize the land-use structure and industrial structure. The optimization can be conducted from the four aspects of land use structure, investment and labor, ESV, and undesirable environmental output and industry structures. There are certain differences in the direction and magnitude of the adjustment.

## Figures and Tables

**Table 1:** List of cities of MRYR.

City Circle	City	Area (km^2^)	GDP ( CNY 100 Million)	City Circle	City	Area (km^2^)	GDP (CNY 100 Million)
Poyang Lake City Circle	Fuzhou	18,799	1573	Wuhan City Circle	Ezhou	1594	1005
	Ji’an	25,373	2169		Huanggang	17,457	2170
	Jingdezhen	5261	957		Huangshi	4583	1641
	Jiujiang	19,798	3241		Qianjiang	558	765
	Nanchang	7402	5746		Tianmen	440	617
	Pingxiang	3831	964		Wuhan	8494	15,616
	Shangrao	22,791	2624		Xiantao	598	828
	Xinyu	3178	1001		Xianning	10,033	1525
	Yichun	18,669	2790		Xiaogan	8910	2194
	Yingtan	3560	983				
Chang-Zhu-Tan City Circle	Changde	18,910	3749	Xiang-Jing-Yi City Belt	Jingmen	12,404	1906
	Hengyang	15,303	3509		Jingzhou	14,067	2369
	Loudi	8119	1680		Xiangyang	19,728	4602
	Xiangtan	5008	2343		Yichang	21,230	4261
	Yiyang	12,320	1853				
	Yueyang	14,858	4002				
	Changsha	11,816	12,143				
	Zhuzhou	11,272	3106				

Data sources: “Bulletin of Statistics for national economic and social development (2020)” of cities in China; *Hubei Statistical Yearbook (2020); Hunan Statistical Yearbook (2020); Jiangxi Statistical Yearbook (2020).*

**Table 2:** Index selection of input and output.

Indicators	Units	Mean	Max	Min	Std. dev.
Input indicators					
Land resources [23,28]					
Farming land	hm^2^	417,021.27	991,976.67	76,749.21	241,549.89
Construction land [7]	hm^2^	39,760.90	118,698.03	7330.41	23,151.40
Other land	hm^2^	671,166.13	1,815,773.94	21,200.49	510,568.82
Capital resource [28]					
Investment in fixed assets [7,19,29,37]	CNY 10 thousand	11,456,262.07	95,856,748.59	49,835.00	16,168,281.28
Labor resource [28,38]					
Number of people employed [7]	10 thousand persons	233.03	603.79	47.20	127.93
Output indicators					
Ecological value [39]					
ESV	CNY 10 thousand	1,558,322.27	4,033,055.02	128,802.86	1,109,511.41
Economic value [23,28,38]					
First industry	CNY 100 million	146.38	513.01	10.85	121.54
Secondary industry	CNY 100 million	642.72	5557.47	21.30	890.34
Tertiary industry	CNY 100 million	615.14	9656.40	20.80	1149.71
Environmental pollution [23,28]					
Industrial sulfur dioxide emission	ton	35,338.50	133,442.00	408.00	29,130.17
Industrial wastewater discharge	10 thousand ton	6278.34	40,661.00	229.07	6139.99

Data sources: *Urban Statistical Yearbook of China* (2001–2021); *China Statistical Yearbook* (2001–2021); *Hubei Statistical Yearbook* (2001–2021); Land cover data of 3 central provinces from “Geospatial Information Platform of Chinese Academy of Sciences”.

**Table 3:** Summary of slacks of input and output factors of the city groups.

	PYL (2000)	WH (2000)	XJY (2000)	CZT (2000)	PYL (2005)	WH (2005)	XJY (2005)	CZT (2005)	PYL (2010)	WH (2010)
FAR	17,565.81	12,141.54	356,716.81	115,491.05	143,545.48	203,402.96	647,902.37	111,301.54	282,065.76	24,217.07
CON	7943.45	18,241.37	23,220.75	2165.35	56,315.08	46,447.90	47,722.22	9613.66	54,778.64	38,856.25
ECO	103,009.95	29,752.10	0.00	36,812.74	380,220.94	227,122.64	243,699.72	30,202.43	76,967.56	0.00
INV	52,359.85	193,986.57	533,404.58	427,900.74	45,640.84	588,068.49	0.00	606,673.91	0.00	475,709.54
LAB	52.35	56.67	0.00	151.23	278.75	158.32	15.82	218.71	107.35	150.42
ESV	2,161,125.56	2,076,313.64	1,759,700.52	449,768.73	1,204,294.50	60,810.98	268,904.44	80,595.93	1,286,006.34	91,228.77
WAS	11.47	47.02	0.00	159.21	83.41	6.30	5.65	85.83	27.59	12.34
SUL	135.22	280.56	143.14	466.28	1200.63	112.50	202.52	425.99	313.34	121.93
FIR	63.17	102.72	12.14	38.74	99.71	30.38	29.13	93.83	158.70	281.43
SEC	156.59	0.00	13.70	68.82	177.20	337.17	33.40	70.52	227.72	127.38
TER	105.61	39.46	3.90	187.36	125.19	64.81	0.75	0.69	2335.83	307.32
	XJY (2010)	CZT (2010)	PYL (2015)	WH (2015)	XJY (2015)	CZT (2015)	PYL (2020)	WH (2020)	XJY (2020)	CZT (2020)
FAR	266,797.90	26,065.09	479,828.46	366,794.66	671,361.84	129,560.57	21,979.83	100,085.56	336,390.11	49,779.53
CON	33,081.76	13,080.42	58,225.20	48,923.33	15,792.13	0.00	15,167.92	15,542.98	22,773.50	3786.50
ECO	77,843.26	15,710.24	6259.88	0.00	0.00	14,871.61	2758.74	327,514.55	866.28	4449.11
INV	407,062.82	1,875,769.44	5,692,966.97	0.00	0.00	599,826.50	5,822,738.28	6,206,309.79	3,606,866.86	7,238,377.27
LAB	72.94	163.01	201.55	237.70	10.69	121.63	42.35	101.69	2.29	36.40
ESV	640,261.14	499,817.29	374,269.74	62,219.58	303,286.41	359,972.31	2,699,105.25	661,178.44	2,066,729.26	3,921,942.00
WAS	0.73	18.06	25.31	6.58	8.41	5.34	1.68	1.53	0.43	1.55
SUL	5.09	164.73	257.11	101.04	32.77	97.04	17.71	6.08	1.63	8.47
FIR	45.69	125.24	350.45	16.38	0.00	126.49	15.94	379.30	319.07	762.86
SEC	222.26	698.01	314.30	955.92	138.91	1198.40	19.21	1714.26	225.22	3759.41
TER	139.19	408.92	3874.85	2424.61	2212.25	2041.21	13.29	5138.07	294.33	5933.15

## Data Availability

The data presented in this study are available on request from the corresponding author.
